# The effectiveness of a monetary incentive offer on survey response rates and response completeness in a longitudinal study

**DOI:** 10.1186/s12874-017-0353-1

**Published:** 2017-04-26

**Authors:** Shengchao Yu, Howard E. Alper, Angela-Maithy Nguyen, Robert M. Brackbill, Lennon Turner, Deborah J. Walker, Carey B. Maslow, Kimberly C. Zweig

**Affiliations:** World Trade Center Health Registry, New York City Department of Health and Mental Hygiene, 125 Worth Street, 10th Floor, New York City, NY 10013 USA

**Keywords:** Incentives, Response rates, Non-response bias, Questionnaires, Return rates, Survey response, Data collection

## Abstract

**Background:**

Achieving adequate response rates is an ongoing challenge for longitudinal studies. The World Trade Center Health Registry is a longitudinal health study that periodically surveys a cohort of ~71,000 people exposed to the 9/11 terrorist attacks in New York City. Since Wave 1, the Registry has conducted three follow-up surveys (Waves 2–4) every 3–4 years and utilized various strategies to increase survey participation. A promised monetary incentive was offered for the first time to survey non-respondents in the recent Wave 4 survey, conducted 13–14 years after 9/11.

**Methods:**

We evaluated the effectiveness of a monetary incentive in improving the response rate five months after survey launch, and assessed whether or not response completeness was compromised due to incentive use. The study compared the likelihood of returning a survey for those who received an incentive offer to those who did not, using logistic regression models. Among those who returned surveys, we also examined whether those receiving an incentive notification had higher rate of response completeness than those who did not, using negative binomial regression models and logistic regression models.

**Results:**

We found that a $10 monetary incentive offer was effective in increasing Wave 4 response rates. Specifically, the $10 incentive offer was useful in encouraging initially reluctant participants to respond to the survey. The likelihood of returning a survey increased by 30% for those who received an incentive offer (AOR = 1.3, 95% CI: 1.1, 1.4), and the incentive increased the number of returned surveys by 18%. Moreover, our results did not reveal any significant differences on response completeness between those who received an incentive offer and those who did not.

**Conclusions:**

In the face of the growing challenge of maintaining a high response rate for the World Trade Center Health Registry follow-up surveys, this study showed the value of offering a monetary incentive as an additional refusal conversion strategy. Our findings also suggest that an incentive offer could be particularly useful near the end of data collection period when an immediate boost in response rate is needed.

## Background

Achieving adequate response rates is an ongoing challenge in survey research. Trend studies suggest that survey participation has decreased over time and further studies have since sought to better understand the drivers of survey nonparticipation [1, 2]. One of the reasons has to do with the increasing number of surveys and research studies being introduced in many fields. Other reasons include the rise of telemarketing, concerns about privacy and confidentiality, and a general decline in volunteerism [1, 3]. For longitudinal or cohort studies that re-survey the same population every few years, survey participation decreases with each follow-up because of waning interest in the survey subject matter [4]. The World Trade Center (WTC) Health Registry, which surveys a sizable cohort to identify and track long term health impact of the September 11, 2001 (9/11) terrorist attacks in New York City, is now facing the challenge of maintaining a response rate comparable to the previous surveys.

Efforts to increase response rates in surveys have been well documented, particularly for mailed questionnaires and telephone surveys. Strategies have generally been categorized and identified by technique and timing. Common technique strategies include enhancing the survey’s visual appeal and improving the delivery process for participants (i.e., pre-paid return envelopes). Timing efforts in boosting response rate usually refer to following up with participants via telephone calls and/or mailings before and after survey launch [5–7].

A third category of response rate enhancing strategies are incentives. Monetary incentives, either prepaid or promised, have long been utilized as a method to increase response rates [2]. A systematic review of 49 studies using mailed questionnaires found that a monetary incentive doubled the odds of returning a completed or partially completed questionnaire [6]. Early studies also reported the positive impact of monetary incentives on increasing response rate, particularly with increased contact and follow up with participants [6, 8]. While the general results suggest that prepaid incentives are the most effective in increasing response rates, promised incentives may also yield successful outcomes [5, 6, 9]. In addition, the dose-effect relationship between incentive value and response rate remains unclear. In a review of ten meta-analyses, five analyses found evidence for a positive linear relationship; however, these findings are inconclusive since these analyses did not control for factors such as survey mode and timing. Considering that meta-analyses typically examine a number of studies conducted in different periods of time, the value of the incentives in older studies may not be comparable to more recent studies [10]. The impact of survey incentives on reducing response bias is conflicting. Some studies have shown that incentives result in a higher percentage of lower socioeconomic respondents while other studies demonstrate a positive impact on the representation of the target population [11]. The effect of incentives on response completeness or item nonresponse, responses to sensitive questions, and length of answers, is also ambiguous [12]. These differences mainly result from size of incentive, the target population, and the mode of data collection (e.g., mailed questionnaire vs. mobile or web-administered surveys) [7, 13].

Since the baseline or Wave 1 survey in 2003–04, the WTC Health Registry has conducted three follow-up surveys (Waves 2, 3, and 4) every 3 to 4 years. Due to substantial efforts made on maintaining the Registry cohort through various communications with enrollees over the years, the overall attrition rate of the Registry cohort is low. For instance, only 4% of the entire cohort was not eligible for the Wave 4 adult survey because of deaths or withdrawals. The Registry has also utilized the aforementioned conventional strategies during all survey waves to increase survey participation, such as prepaid return envelopes, email and postcard reminders, telephone outreach, and door-to-door outreach. These efforts have resulted in response rates of 68 and 63% for Wave 2 and Wave 3, respectively. In supplementing these endeavors and strategies, a monetary incentive was offered in the most recent Wave 4 survey. More specifically, an incentive experiment was conducted among non-respondents five months after the survey had launched. In utilizing an incentive strategy for the first time, this study aims to evaluate the effectiveness of incentive use in maintaining and improving the response rate via refusal conversion, and to assess whether or not incentive use compromises response completeness. The objectives of this study are to 1) compare the likelihood of responding to a survey for those who received an incentive offer to those who did not; and 2) examine whether an incentive notification increases response completeness among those who return surveys.

This study serves to expand and contribute to the existing literature in incentive use in several ways. First, relatively few studies to our knowledge have focused on the impact of incentive use in longitudinal studies. The context in which the incentive experiment was executed in this study is unique as it was conducted among a group of survey non-respondents enrolled in a long-running longitudinal study. Second, this study assesses the impact of the incentive on refusal conversion with particular attention to survey mode, as an incentive offer may increase response rates differently between paper and web administered survey respondents [14]. Third, this study also examines timing of responding to a survey after the incentive is introduced and how the incentive may affect response completeness for returned surveys.

## Methods

### Setting

Study participants were from the WTC Health Registry. Registry enrollees who had not yet responded to the Wave 4 health survey about five months into data collection were randomized into two groups: 1) an incentive group received incentive offer notifications sent via postcard reminder, in addition to an email reminder with a personalized survey link if email address were available, both containing a promised $10 cash incentive upon returning a completed survey; 2) a comparison group also received postcard and/or email reminders as did the incentive group, however with no mention of an incentive offer in any of the communications.

All participants gave verbal informed consent to participate in the WTC Health Registry at the time of enrollment in 2003–04. The US Centers for Disease Control and Prevention and the New York City Department of Health and Mental Hygiene institutional review boards approved the Registry protocol, including use of the data.

### Randomization

The study sample was limited to English speaking enrollees from single-enrollee households, to avoid potential confusion upon receiving different communications within the same household and to avoid operational complexity. Of the 35,421 non-respondents, we randomly assigned 2174 enrollees to the group not receiving incentive offer notifications, and 33,247 enrollees to the group receiving the incentive notification. The number of enrollees in each group was determined to be sufficient after we calculated the sample size necessary to distinguish a pre-determined difference between two proportions of survey respondents, where one group size was pre-set to be eighteen times that of the other. In particular, we chose a type I error of α = 0.05, a power of 80%, and group response rates of 0.25 and 0.35. We chose to use a comparatively small non-incentive group because while we wanted to be able to assess how well the incentive worked, we did not want to unnecessarily reduce our response rate by limiting the potential benefits of the incentive to a smaller group of non-respondents.

### Data collection

The WTC Health Registry Wave 4 survey was launched in March 2015. Five months into survey data collection, 37% of the eligible enrollees had completed and returned their surveys following two rounds of paper survey mailings, ten email reminders with survey links, and four rounds of postcard reminders. During the following three months, an incentive experiment was conducted among non-respondents to learn whether or not a promised monetary incentive would boost the response rate five months after the survey had launched. We did not offer a prepaid incentive or an incentive from the beginning of the study due to budget constraints. During the three-month incentive experiment period, reminder phone calls and door-to-door outreach were also conducted concurrently, in addition to email and postcard reminders that were sent frequently throughout the data collection period.

### Outcome measures

We measured the impact of incentive notification in two ways. First, survey response was defined as receipt of a returned Wave 4 health survey, either online or by mail. Second, response completeness among those who returned a survey was measured for two types of variables: a) for all five multi-item questions in the survey, by the number of items completed, and b) for all dichotomous questions, by whether or not an answer was provided.

### Covariates

Five sociodemographic variables, including sex, age on 9/11, race/ethnicity, total household income in 2002 and education, were included in the analyses to capture characteristics of participants. Other covariates included in the analysis were two additional response rate boosting strategies that were employed simultaneously with incentive notification: reminder phone calls and door-to-door outreach. Wave 4 non-respondents with valid telephone numbers, who had completed all three previous surveys, received reminder calls to complete the survey. In addition, door-to-door outreach, or home visit, was conducted among non-respondents who resided in lower Manhattan housing projects, and among residents in other areas within the five boroughs of New York City whose home addresses formed a cluster geographically, and among very few non-respondents who completed all three previous surveys and resided in lower Manhattan. Another covariate was the number of surveys completed out of the three previous surveys.

### Statistical analysis

Sociodemographic characteristics and other features of the study sample were described by incentive group. We then predicted the likelihood of returning a survey when offering an incentive while adjusting for covariates in logistic regression models. In this stage, the likelihood of returning a survey was assessed for the entire sample and for two sub-groups, one group receiving incentive notification via only postcard due to lack of email address while another group receiving incentive notification via both postcard and email. The purpose of running two separate models for postcard group and postcard-email combined group was to assess if the mode of communication interacted with the effect of incentive in boosting response rate. We also examined the impact of the incentive for the short period immediately after the incentive offer notification, before sending additional communications such as email and postcard reminders, to understand how quickly and how well an incentive offer alone could work before further communications.

Finally, of the returned surveys, we examined whether or not enrollees receiving incentive notification provided more complete responses than those who were not offered incentives, for the two types of variables mentioned above: a) for five multi-item questions, we used negative binomial regression to determine the ratio of the average number of questions answered in the two groups, b) for all dichotomous questions, we used logistic regression to determine the odds ratio of answering the question in the two groups.

## Results

Among 35,421 enrollees in the study sample, the majority were male (61%), aged 25–44 on 9/11 (60%), and non-Hispanic White (60%) (see Table 1). This sample had relatively high income and education, with 66% having household income above $50,000 in year 2002 and over 75% having college or post-graduate education. About 25% of the sample were on the targeted reminder call list and 6% were on the door-to-door outreach visit list. More enrollees had participated in all three previous surveys (38%), compared to those who participated in only one (32%) or two (30%) previous surveys. Table 1 clearly shows that the study sample was randomly distributed by incentive group across all covariates considered in this study.

**Table 1:** Sample characteristics by incentive group

	Total		Incentive group	
	*N* = 35,421	%	Yes (*N* = 33,247) %	No (*N* = 2174) %
Sex				
Female	13,752	38.8	38.8	39.5
Male	21,669	61.2	61.2	60.5
Age at 9/11				
< 18	692	2.0	2.0	2.1
18–24	2921	8.3	8.3	8.3
25–44	21,364	60.5	60.4	61.8
45–64	9730	27.5	27.6	26.2
65+	625	1.8	1.8	1.6
Race				
Non-Hispanic white	21,416	60.5	60.5	59.7
Non-Hispanic black	5487	15.5	15.5	15.3
Hispanic	4805	13.6	13.5	14.2
Asian	2044	5.8	5.8	5.8
Multi-racial	696	2.0	1.9	2.4
Other	973	2.8	2.8	2.6
Total household Income in 2002, $				
< 25,000	3077	9.9	9.8	10.4
25,000– < 50,000	7405	23.8	23.8	23.7
50,000– < 75,000	6919	22.2	22.2	22.5
75,000– < 150,000	10,397	33.4	33.4	33.0
> =150,000	3359	10.8	10.8	10.5
Education				
High school and below	8537	24.6	24.5	24.9
College	9030	26.0	26.0	25.2
Post-graduate	17,205	49.5	49.5	50.0
Reminder call				
Yes	8775	24.8	24.7	25.4
No	26,646	75.2	75.3	74.6
Door to door outreach				
Yes	2166	6.1	6.1	6.4
No	33,255	93.9	93.9	93.6
Number of previous surveys completed				
Three	13,435	37.9	37.9	38.0
Two	10,666	30.1	30.1	29.7
One	11,320	32.0	31.9	32.3

A higher proportion of enrollees from the incentive offer notification group returned a survey than those from the non-incentive group (19.4% vs. 16.5%, see Table 2). Based on these response rates, we estimated that for the entire study sample, 1033 more surveys would have been received with an incentive offer as compared to no such offer during the 3-month period of incentive experiment. That was equivalent to an 18% increase over the number of surveys we would have received without an incentive.

**Table 2:** Incentive and likelihood of returning a survey for study sample

	Returned survey				Likelihood of returning	
	No (N, %)		Yes (N, %)		AOR^a^	95% CI^b^
	28,602	80.7	6819	19.3		
Incentive group						
Yes	26,787	80.6	6460	19.4	1.3	(1.1, 1.4)
No	1815	83.5	359	16.5	Ref	1
Sex						
Female	11,023	80.2	2729	19.8	1.2	(1.1, 1.2)
Male	17,579	81.1	4090	18.9	Ref	1
Age at 9/11						
< 18	596	86.1	96	13.9	1.4	(1.1, 1.9)
18–24	2500	85.6	421	14.4	0.9	(0.8, 0.9)
25–44	17,396	81.4	3968	18.6	Ref	1
45–64	7527	77.2	2223	22.9	1.2	(1.1, 1.3)
65+	518	82.9	107	17.1	0.8	(0.6, 1.0)
Race						
Non-Hispanic white	16,756	78.2	4660	21.8	Ref	1
Non-Hispanic black	4671	85.1	816	14.9	0.8	(0.7, 0.9)
Hispanic	4019	83.6	786	16.4	0.9	(0.8, 1.0)
Asian	1713	81.8	331	16.2	0.8	(0.7, 0.9)
Multi-racial	584	83.9	112	16.1	0.8	(0.6, 0.9)
Other	859	88.3	114	11.7	0.7	(0.5, 0.9)
Total household Income in 2002, $						
< 25,000	2635	85.6	442	14.4	Ref	1
25,000– < 50,000	6156	83.1	1249	16.9	1.1	(0.9, 1.2)
50,000– < 75,000	5472	79.1	1447	20.9	1.2	(1.1, 1.4)
75,000– < 150,000	8131	78.2	2266	21.8	1.2	(1.0, 1.3)
> =150,000	2636	78.5	723	21.5	1.1	(0.9, 1.3)
Education						
High school and below	7186	84.2	1351	15.8	Ref	1
College	7305	80.9	1725	19.1	1.1	(1.1, 1.3)
Post-graduate	13,522	78.6	3683	21.4	1.3	(1.2, 1.4)
Reminder call						
Yes	5528	63.0	3247	37.0	1.3	(1.2, 1.4)
No	23,074	86.6	3572	13.4	Ref	1
Door to door outreach						
Yes	1995	92.1	171	7.9	0.3	(0.3, 0.4)
No	26,607	80.0	6648	20.0	Ref	1
Number of previous surveys completed						
Three	8733	65.0	4702	35.0	7.7	(6.8, 8.6)
Two	9151	85.8	1515	14.2	2.8	(2.5, 3.1)
One	10,718	94.7	602	5.3	Ref	1

^a^AOR: adjusted odds ratio and was adjusted for all factors listed in this table

^b^95% CI: 95% confidence interval

After adjusting for sociodemographic characteristics and other covariates, incentive notification increased the odds of returning a survey significantly by 1.3 (95% CI: 1.1, 1.4, see Table 2). The likelihood of enrollees responding to a survey was also associated with being female, younger, non-Hispanic White, having a higher household income and higher education. Receiving reminder phone calls also increased the odds of returning a survey by 1.3 (95% CI: 1.2, 1.4). However, visiting homes of those living in Lower Manhattan housing projects or clustered in other areas of New York City reduced their likelihood of returning a survey by 70%, relative to those whose homes were not visited (AOR = 0.3, 95% CI: 0.3, 0.4). We suspect that the unexpected negative impact of home visit on response was related to how these homes were selected in this study. Participation in earlier surveys was a strong predictor of responding to a Wave 4 survey. As compared to those who only completed Wave 1 survey, those who completed all three previous surveys were 7.7 times more likely to respond to Wave 4 survey (95% CI: 6.8, 8.6); and participating in two of the three previous surveys increased the odds of returning a Wave 4 survey by 2.8 (95% CI: 2.5, 3.1). In pooled models, there were no statistically significant interactions between incentive and number of previous surveys completed in their effects on the likelihood of returning a survey (results not shown). Furthermore, we ran stratified models by number of previous waves completed to determine whether or not the effectiveness of the incentive was similar across three levels of previous survey participation. Our analyses using Breslow-Day test indicated that the incentive and survey response association did not differ statistically significant among those who had completed one, two, or all three of the previous surveys (results not shown).

Among the 35,421 enrollees in the study sample, the Registry was able to communicate with about 51% of them via email, while the remaining 49% could only be reached by mail due to unavailable email addresses. Incentive offer notifications were therefore sent to the two groups by email and postcard respectively. Table 3 presents the major differences in likelihood of responding to a survey for these two groups. Enrollees aged under 18 years old (currently under 33 years old) were more likely to complete their surveys relative to those older (aged 25–44 on 9/11) in the email group, while no such significant age effect was observed in the postcard reminder group. Non-Hispanic Whites were more likely to respond to the survey compared to other races/ethnicities among the email group; in contrast, among the postcard group, being White increased the odds relative to being non-Hispanic Black only. In the postcard group, those who received phone call outreach were more likely to respond to the survey (AOR = 1.6, 95% CI: 1.4, 1.8), but no significant impact was observed for those receiving calls in the email group.

**Table 3:** Incentive and likelihood of returning a survey by communication group

	Email group^a^		Postcard group	
	Likelihood of returning		Likelihood of returning	
	AOR^b^	95% CI^c^	AOR^b^	95% CI^c^
Incentive group				
Yes	1.3	(1.1, 1.5)	1.3	(1.0, 1.6)
No	Ref	1	Ref	1
Sex				
Female	1.1	(1.1, 1.2)	1.2	(1.1, 1.4)
Male	Ref	1	Ref	1
Age at 9/11				
< 18	1.7	(1.2, 2.3)	0.9	(0.5, 1.5)
18–24	0.8	(0.7, 1.0)	0.8	(0.6, 1.0)
25–44	Ref	1	Ref	1
45–64	1.3	(1.2, 1.4)	1.2	(1.0, 1.3)
65+	1.3	(0.9, 1.9)	0.7	(0.5, 1.1)
Race				
Non-Hispanic white	Ref	1	Ref	1
Non-Hispanic black	0.8	(0.8, 0.9)	0.8	(0.7, 0.9)
Hispanic	0.9	(0.8, 1.0)	1.1	(0.9, 1.3)
Asian	0.8	(0.7, 1.0)	0.8	(0.6, 1.1)
Multi-racial	0.7	(0.5, 0.9)	0.8	(0.6, 1.3)
Other	0.7	(0.5, 1.1)	0.7	(0.4, 1.3)
Total household Income in 2002, $				
< 25,000	Ref	1	Ref	1
25,000– < 50,000	1.0	(0.9, 1.2)	1.0	(0.8, 1.3)
50,000– < 75,000	1.1	(0.9, 1.3)	1.2	(0.9, 1.4)
75,000– < 150,000	1.0	(0.9, 1.2)	1.2	(0.9, 1.5)
> =150,000	1.0	(0.8, 1.2)	1.1	(0.8, 1.4)
Education				
High school and below	Ref	1	Ref	1
College	1.0	(0.9, 1.2)	1.1	(0.9, 1.3)
Post-graduate	1.1	(1.0, 1.3)	1.2	(1.1, 1.4)
Reminder call				
Yes	1.1	(1.0, 1.2)	1.6	(1.4, 1.8)
No	Ref	1	Ref	1
Door to door outreach				
Yes	0.3	(0.2, 0.4)	0.5	(0.3, 0.6)
No	Ref	1	Ref	1
Number of previous surveys completed				
Three	6.0	(5.1, 6.9)	6.9	(5.8, 8.3)
Two	2.4	(2.1, 2.7)	2.5	(2.1, 3.0)
One	Ref	1	Ref	1

^a^Email group: this group also received postcard and mail communications as the postcard group

^b^AOR: adjusted odds ratio and was adjusted for all factors listed in this table

^c^95% CI: 95% confidence interval

Despite the differences presented above between the postcard group and email group, the impact of the incentive offer was almost the same for enrollees in both groups, with the odds of returning a survey being 1.3 times higher for those who received an incentive offer notification than those who did not.

To assess how much the incentive offer increased the likelihood of responding to a survey without additional outreach efforts, we ran the same logistic regression model for the 12 days immediately after the incentive notification, before additional reminders were sent. An incentive offer increased the odds of responding to a survey by 1.5 (95% CI: 1.1, 2.2) (results not shown in Tables) in the first 12 days, slightly higher than the odds for the entire duration of the incentive experiment period (Table 2, AOR = 1.3, 95% CI: 1.1, 1.4).

Among those who returned surveys during the incentive experiment period (*N* = 6819), we ran a thorough analysis of response completeness across all survey items. Our results showed that receiving an incentive offer did not significantly affect response completeness, as defined earlier. For example, for all five multi-item questions in the survey, the ratio of the average number of questions answered between the incentive and non-incentive groups was not statistically or substantially different from one. For all sixty-one but two dichotomous questions, the odds ratio of answering a question was not statistically different from one (i.e. the 95% confidence interval included one). For the two dichotomous questions where the odds ratio was different from one, the proportion answered was not substantially different for the incentive and non-incentive groups (e.g. 95% vs. 93%). In Table 4, we presented the negative binomial regression results for the five multi-item questions (physical health, posttraumatic stress disorder, depression, psychological distress, and social support), and the logistic regression results for five selected (out of sixty-one) dichotomous yes/no questions. Using the 22-item physical health question as an example, the average number of items answered for the incentive and non-incentive groups was not significantly different, 20.5 and 20.4 respectively (see Table 4). For the cancer question (yes/no), the odds ratio for providing an answer for the incentive group was 1.5 relative to the non-incentive group, but the association was not significant (AOR = 1.5, 95% CI: 0.7, 3.2).

**Table 4:** Comparison of response completeness by incentive group

	Incentive group	Non-incentive group	Association	
Multi-item questions (N of items)	Average number of items answered		Rate ratio	95% CI^a^
Physical health (22)	20.5	20.4	0.9	(0.7, 1.3)
Posttraumatic stress disorder (17)	16.7	16.8	1.3	(0.7, 2.4)
Depression (8)	7.8	7.8	0.8	(0.4, 1.5)
Psychological distress (6)	5.9	5.8	0.7	(0.3 1.5)
Social support (5)	4.9	4.9	0.8	(0.3, 2.2)
Dichotomous questions	Proportion answered		Odds ratio	95% CI^a^
Cancer	0.985	0.978	1.5	(0.7, 3.2)
Cough	0.984	0.986	1.2	(0.5, 2.9)
Health insurance	0.987	0.989	1.2	(0.4, 3.2)
Smoking	0.986	0.983	0.8	(0.4, 1.9)
Weight	0.979	0.994	3.7	(0.9, 15.0)

^a^95% CI: 95% confidence interval

## Discussion

Fourteen to fifteen years after 9/11 disaster, 55% of the eligible enrollees responded to the WTC Health Registry’s Wave 4 survey, and the response rate for those who completed all three previous surveys reached 78%. The results of this study show that a $10 incentive offer was effective in increasing response rates in the Wave 4 survey. Specifically, our findings showed that a $10 incentive offer was useful in refusal conversion, i.e., converting the initially reluctant participants to respond, and to respond earlier. A 30% increase in the likelihood of responding to a survey, or an 18% increase in the number of returned surveys during the 3-month incentive experiment period demonstrated the value of offering a monetary incentive in this longitudinal health study. In addition, this study showed that an incentive offer could still improve survey response rate even when it was offered among non-respondents who did not respond to rounds of prior reminders, and even when it was offered as promised compensation upon a returned survey. We also found that the association between the incentive and survey response did not vary significantly by socio-demographic groups, such as sex, age, race, income, or education (results not presented). This finding provides assurance that there was no differential effect of the incentive on refusal conversion in this population. The magnitude of the incentive effect on response rates found in this study was similar to that from a previous study among recent U.S. veterans [15], in which the entire sample was offered either promised or prepaid incentive from the beginning of the study.

The effect of incentive offer found in this study was immediate and independent of the impact of other response boosting strategies, and this effect was more consistent across groups (email versus postal contact) than other response rate enhancement strategies. For example, as we found in this study, placing reminder calls did increase the odds of responding to the survey among enrollees with whom we contacted only through postal services (e.g., postcard and letters) (AOR = 1.6, 95% CI: 1.4, 1.8, see Table 3) while for enrollees who also received email communications, the effect of reminder phone calls was not significant. However, we observed that the impact of incentive offer was consistent and the odds of increasing response were the same for the two groups with different means of communication. It was clear that even five months into data collection, non-respondents read reminders sent by both postcard and email, and they noted and responded to the incentive offer in the communications.

To further disentangle the potential interactive effect of incentive and other response boosting strategies, such as additional email and postcard reminders which we did not control for in our analytical model, we assessed the impact of incentive for the first 12 days following the initial incentive offer, before further reminders were sent. Our results showed that incentive offer increased the likelihood of responding to the survey by 50% during this period (AOR = 1.5, 95% CI: 1.1, 2.2). It is apparent that the incentive offer produced an early and prompt response among many Registry enrollees during this time without requiring further reminders. This immediate and significant effect of incentive offer convincingly suggests that providing an incentive can be an effective additional refusal conversion strategy to increase response rates, particularly near the end of data collection period.

One concern with using incentive in health surveys is that it may bias the response and affect data quality through compromised estimates of disease incidence and prevalence. In this study we examined survey data from returned surveys and estimated if the incentive offer affected response completeness. Our results did not reveal any significant differences on response completeness between those who received an incentive offer and those who did not. Although response completeness captures only one of the many aspects of data quality, this finding provided a level of confidence in using an incentive to boost response rate among Registry enrollees without introducing bias in response completeness. Another concern, as noted in the existing literature, is the ethical issue in offering incentives to some participants and not to others. However, the number of enrollees assigned to non-incentive group in our study was small, and the incentive experiment only lasted for three months. Everyone in this non-incentive group was offered the same incentive after the three months of experiment period. In addition, to address these ethical considerations, all enrollees in the comparison group who completed the survey during the experimental period also received the incentive.

## Conclusions

In the face of the growing challenge of maintaining a high response rate for the WTC Health Registry follow-up surveys, this study showed the value of offering a monetary incentive as an additional refusal conversion strategy. Our findings also suggest that an incentive offer could be particularly useful near the end of the data collection period when an immediate boost in response rate is needed.

## Abbreviations

AOR: Adjusted odds ratio
CI: Confidence interval
WTC: World Trade Center
